# Case report: a rapid review approach used by the UK National Screening Committee to inform recommendations on general population screening for vasa praevia

**DOI:** 10.1186/s13643-019-1244-9

**Published:** 2019-12-29

**Authors:** Saoirse Leonard, Amy Buchanan-Hughes, Anna Bobrowska, Cristina Visintin, John Marshall

**Affiliations:** 1 Costello Medical, Cambridge, UK; 2 UK National Screening Committee, London, UK

**Keywords:** Rapid review, Population screening, Vasa praevia, Velamentous cord insertion

## Abstract

**Background:**

The UK National Screening Committee (UK NSC) reviews evidence about existing or potential population screening programmes using rapid review products called evidence summaries. We provide a case report as an example of how rapid reviews are developed within the UK NSC’s process, consider how the quality of rapid reviews should be assessed and ask whether the rapid review was an appropriate tool to inform the UK NSC’s decision-making process.

**Methods:**

We present the rapid review approach taken by the commissioner and the reviewers to develop an evidence summary for vasa praevia (VP), which the UK NSC reappraised as part of its 3-yearly cycle for conditions where screening is currently not recommended. We apply the AMSTAR 2 quality appraisal checklist for systematic reviews, the Preferred Reporting Items for Systematic Reviews and Meta-Analyses (PRISMA) checklist and a published checklist of items to consider with a rapid review approach. As UK NSC evidence summaries do not include meta-analyses, any related AMSTAR 2 or PRISMA checklist items were considered inapplicable.

**Results:**

The evidence summary was available within the required timelines and highlighted little change from the previous review in terms of key evidence gaps relating to the epidemiology of VP, the screening test and the management pathway. Therefore, the UK NSC concluded that there was insufficient evidence to support a change in its previous recommendation against screening. The evidence summary scored moderately against the applicable AMSTAR 2 and PRISMA checklist items. Against the published checklist of items to consider with a rapid review approach, the evidence summary performed well.

**Conclusions:**

In this case report, the use of a rapid review as part of the UK NSC’s process enabled a pragmatic approach to assessing the overall volume, quality and direction of literature on key questions relating to the viability of a population screening programme for VP. Based on our assessments of this single evidence summary, systematic review quality appraisal tools may undervalue rapid reviews. The validity of the methods used in this case report, as well as the wider generalisability of our insights relating to rapid review practice, reporting and quality assessment, requires analysis of a larger sample of rapid reviews.

## Background

Screening is the process of identifying healthy people who may be at increased risk of a disease or condition. In the UK, the National Screening Committee (NSC) is responsible for reviewing evidence about existing or potential population screening programmes. Evidence reviews are used to advise government ministers and the National Health Service (NHS) on the implementation, continuation and cessation of screening programmes.

Systematic literature reviews (SLRs) are regarded as the gold standard for evidence-informed policy-making because they provide robust, comprehensive and trustworthy appraisals of the evidence on a topic [1]. However, “rapid reviews” have gained increasing attention within policy-making contexts [2]. Rapid reviews aim to modify and expedite the processes and methods used in SLRs without compromising the trustworthiness of the final product [3, 4]. However, evaluation of these products has highlighted concerns related to both conduct and reporting when SLR quality assessment tools are applied to them, in the absence of rapid review-specific quality appraisal tools [5]. It has, however, been shown that end users of rapid reviews do not perceive them as substitutes for SLRs, and value them for a wider range of purposes than providing a definitive answer to a specific research question [3].

For conditions where the current recommendation is not to offer screening, the UK NSC reappraises the evidence base every 3 years using rapid review products called evidence summaries [6].^1^ Evidence summaries serve three purposes: (1) to determine whether there have been significant developments in the evidence base since previous reviews on the topic were conducted, (2) to establish whether current recommendations relating to the screening programme should be reconsidered or reaffirmed and (3) to establish whether further research into the topic is required. This further research could include additional rapid reviews or a full SLR, modelling studies, cost-effectiveness analyses and/or primary research.

**Fig. 1:**
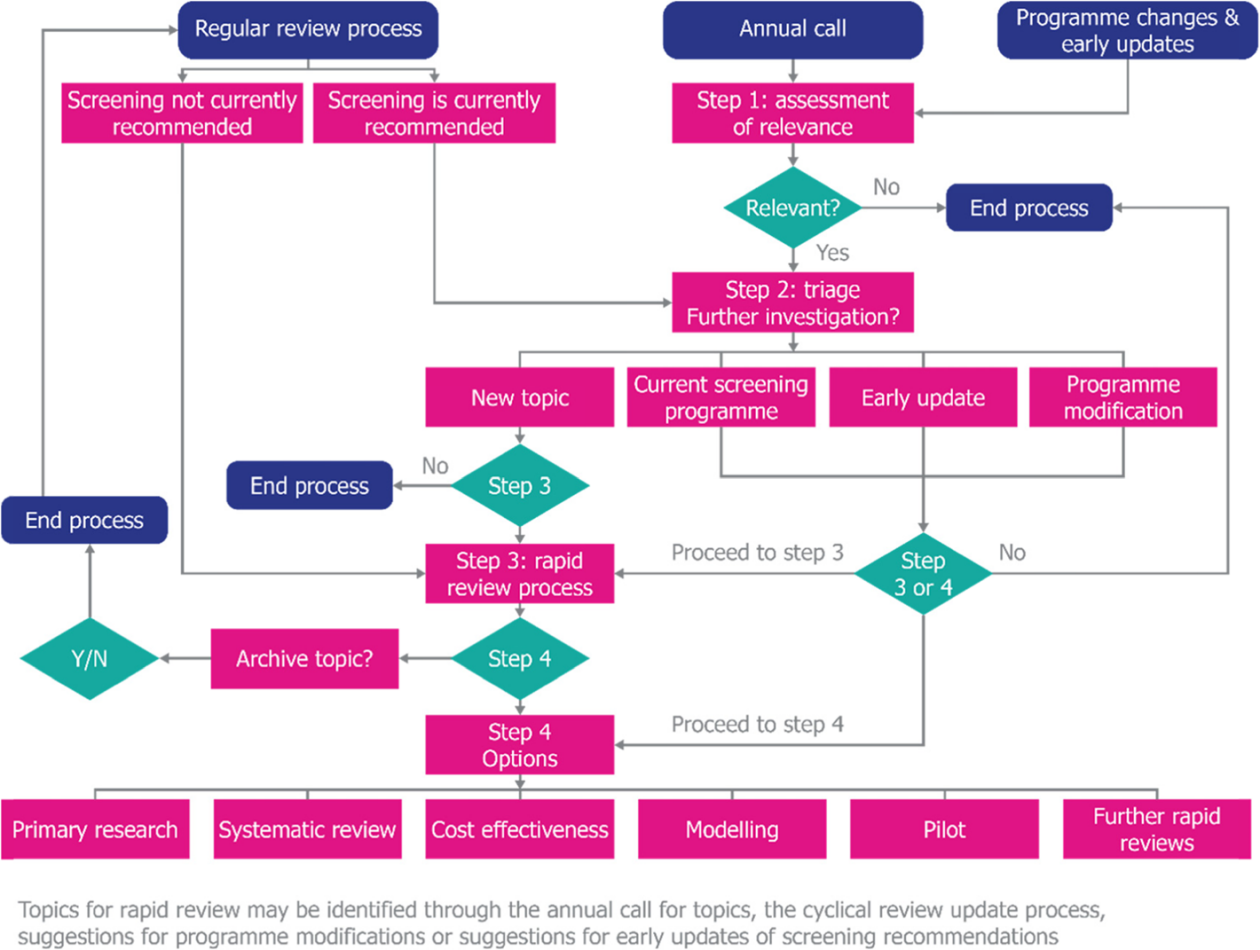
Overall UK NSC evidence review process, indicating how rapid reviews (as evidence summaries) are utilised [8]

The UK NSC assesses all potential screening programmes against a formal list of 20 criteria for appraising their viability, effectiveness and appropriateness. These criteria are structured in line with the principles proposed by Wilson and Jungner [7] and consider a range of issues relating to the condition, the test, the intervention, the screening programme and the implementation of the programme. Evidence summaries do not address all of these domains in a single review; instead, they focus on key questions within a subset of the 20 criteria. The aim is to keep abreast of the evidence relating to over 100 conditions in a way that is proportionate to each. How evidence summaries are utilised within the overall UK NSC evidence review process is shown in Fig. 1.

The UK NSC conducted an appraisal of screening for vasa praevia (VP) as part of the 3-yearly review cycle. VP is a rare but serious obstetric condition where exposed umbilical vessels lie across the cervical opening during pregnancy. If VP is undiagnosed, the blood vessels can rupture during the natural labour process. This can lead to fetal exsanguination, which can be lethal. Ultrasound screening for VP during the second trimester has been proposed [9, 10], using a screening algorithm that includes identification of velamentous cord insertion (VCI), a related marker of risk for VP. The aim of screening is to identify a group of women who would be offered a caesarean section (CS) to prevent the adverse consequences of VP. This evidence summary followed an earlier review on VP conducted for the UK NSC in 2012, which concluded that there was insufficient evidence to recommend universal routine antenatal screening for VP. Detailed methodology and results of the current rapid review can be accessed online [11], and a manuscript reporting the results relating to VCI is under review.

Here, we present the approach taken to develop a UK NSC evidence summary for VP, providing a case report of how rapid reviews are developed within the UK NSC’s evidence review process. We also report the person-time required to produce the evidence summary, discuss the use of systematic review quality appraisal and reporting checklists when applied to this example of a rapid review and consider whether the evidence summary was an appropriate tool to be used by the UK NSC in their decision-making process.

## Methods

The approach taken to developing the evidence summary is detailed below, along with details of the published checklists that we applied to the review product.

### Approach to developing the evidence summary

#### Roles of the commissioning and review teams

The review was commissioned by the UK NSC (CV, JM) and conducted by an external review team who specialise in evidence synthesis (AB, ABH, SL). A summary of the respective roles of the commissioning and review teams on this project is provided in Table 1.

**Table 1:** Respective roles of the commissioning and review teams

Commissioning team	Review team
Identifying and liaising with experts in the areas of antenatal screening and VP throughout the review process	N/A
• Preparing and supplying review brief, outlining: o Aims, background and key questions o Population, Intervention, Comparator and Outcomes (PICO) for each question • Critiquing the proposed review approach, including: o Commenting on draft versions of the review protocol o Providing and coordinating expert input • Providing the evidence summary reporting checklist and evidence summary template	Developing a formal review protocol to meet the review brief provided by the commissioning team, including developing the search strategy
Clarifying queries from the review team regarding the eligibility criteria	• Conducting literature searches • Screening the search results against prespecified eligibility criteria and making final decisions with regard to the eligibility of studies for inclusion in the review • Extracting data from relevant studies and assessing the quality of each study
• Assisting the review team with making judgements as to whether the evidence identified met the UK NSC evidence criteria • Leading discussion of the draft review within the UK NSC reference group structures	Synthesising the data relating to each review question, including making a judgement as to whether the UK NSC’s screening criteria were met or not
Leading the 3-month public stakeholder consultation, including: • Hosting the consultation on the UK NSC website • Analysing the stakeholder input and coordinating appropriate responses and changes to the final product where required	Developing a full report meeting the requirements of the UK NSC’s evidence summary reporting checklist
Preparing the report of the review process for the UK NSC decision-making meeting	Supporting the commissioning team in responding to comments from the public stakeholder consultation

A scoping exercise (including scoping literature searches) and discussion with experts on the previous review within the UK NSC’s reference group structures led to the identification of 9 questions relating to VP and VCI. These were presented to the review team in “Population, Intervention, Comparator and Outcomes” (PICO) format in a brief that also provided background on the topic, details of previous UK NSC reviews and practical points such as the proposed timescale for the evidence summary.

Once the project was underway, regular meetings between the two teams enabled the commissioning team to contribute to discussions on the review protocol and drafts of the report. With regard to study selection, the commissioning team provided the review team with clarifications on the eligibility criteria, but the review team made the final decisions on the eligibility of studies for inclusion in the review. The commissioning team also contributed to discussions with the review team when making judgements as to whether the evidence identified met the UK NSC evidence criteria. Topic area expertise was available as required, and this was most frequently accessed during the protocol development stage and when the review document was being processed within the UK NSC reference group structures.

#### Aims and objectives of the review

The rapid review aimed to identify developments in the evidence base relating to VP since the previous UK NSC review on VP was conducted in 2012, establish whether the current recommendation against screening should be reconsidered and determine whether further research was required.

The 2012 review and subsequent public consultation found that detection of VCI would be an important component of a screening strategy for VP. Most cases of VP are associated with VCI; however, only a minority of pregnancies affected by VCI are also affected by VP, and detection of VCI as part of a VP screening programme would represent a departure from the current approach in UK practice with the potential for over-detection. A set of separate questions relating to VCI were therefore introduced in the current review to explore these issues in more depth.

The previous review on screening for VP was conducted in 2012, before the UK NSC’s evidence summary process was standardised, and the results were not structured in the same way. To enable consistent synthesis of the older and newer evidence and to ensure that relevant evidence on VCI was also identified, the current review included studies published from 2000 onwards. Studies identified in the previous review were therefore re-included and re-analysed in the current review.

The specific, focused questions considered in the review are detailed in Table 2.

**Table 2:** Key questions for the evidence summary and relationship to UK NSC screening criteria

Area	UK NSC criteria	Question(s)
The condition	1. The condition should be an important health problem as judged by its frequency and/or severity. The epidemiology, incidence, prevalence and natural history of the condition should be understood, including development from latent to declared disease, and/or there should be robust evidence about the association between the risk or disease marker and serious or treatable disease	What is the incidence of VP in the UK? If possible, data to be stratified by presence or absence of risk factors
		What percentage of VP cases identified in the second trimester will resolve by late pregnancy?
		What is the risk of adverse perinatal outcomes in pregnancies associated with VP?
		What is the incidence of VCI in the UK? If possible, data to be stratified by presence or absence of risk factors
		What is the risk of adverse perinatal outcomes in pregnancies associated with VCI?
The test	4. There should be a simple, safe, precise and validated screening test	How effective is second-trimester transabdominal sonography for detecting VP?
		How effective is second-trimester transabdominal sonography for detecting VCI?
The intervention	9. There should be an effective intervention for patients identified through screening, with evidence that intervention at a presymptomatic phase leads to better outcomes for the screened individual compared with usual care 10. There should be agreed evidence-based policies covering which individuals should be offered interventions and the appropriate intervention to be offered	What is the most effective management pathway for women with screen-detected VP?
		What is the most effective management pathway for women with screen-detected VCI?

#### Searching the literature

Scoping searches were carried out during the development of the review brief. At the protocol development stage, several decisions were made to streamline the searching process. The overall volume of published literature on VP and VCI is very limited, with fewer than 1000 records identified across key databases (MEDLINE, Embase and the Cochrane Library) even when terms for VP and VCI were used without any filters for dates, study design or outcomes. The reviewers judged that it would be more efficient to search using terms for VP and VCI only rather than developing more specific search strategies for each review question. Taking this approach allowed the database searches and abstract review to be completed within 2 days, in contrast to potentially several weeks if more extensive protocol development had been required.

Hand searching of some excluded SLRs was undertaken, but it was planned that no further supplementary searches would be conducted, such as manual searches of key congress proceedings. Authors were not contacted for further information on studies.

#### Reviewing the literature: eligibility, screening, extraction and quality appraisal

For each question, eligibility criteria were developed to identify studies that could provide the most robust and relevant information. It was prespecified that studies would be prioritised for inclusion in the evidence synthesis based on study design. For example, if a relevant SLR or meta-analysis was identified on a particular question, it would not be considered necessary to include lower quality evidence such as that obtained from retrospective studies. If there were no relevant SLRs or meta-analyses, but an abundance of primary studies on a particular topic, it was planned that the primary studies would be prioritised by study design. For epidemiology studies, longitudinal observational studies would be prioritised over cross-sectional studies. For studies on the performance of screening methods and the effectiveness of management pathways, randomised controlled trials (RCTs) would be given highest priority, followed by interventional non-RCTs, prospective cohort studies, retrospective cohort studies, case control studies and cross-sectional studies. During the rapid review, however, it was not necessary to prioritise any study designs over others; on one topic, an SLR was identified along with all of the primary studies included within it, while on other topics, all primary studies identified were included.

Sifting of each record was performed by a single reviewer; a second independent reviewer provided input in cases of uncertainty and validated a random 20% of the first reviewer’s screening decisions. A single reviewer extracted relevant data from included studies into prespecified extraction tables, which were included as appendices in the evidence summary. A second reviewer then independently verified the extracted information and checked that no relevant information had been missed.

Quality assessment was performed for each study using prespecified checklists suitable for each study design. In SLRs, checklists are sometimes adapted to suit the purposes of the review, but for this rapid review, published checklists were used without adaptation to minimise the time required for protocol development. The published checklists included the following: for epidemiology and prognostic studies, the JBI Critical Appraisal Checklist for Studies Reporting Prevalence Data and the Centre for Evidence Based Medicine Prognostic Studies Critical Appraisal Worksheet, and for diagnostic accuracy studies, the Quality Assessment of Diagnostic Accuracy Studies (QUADAS-2) tool.

#### Synthesis and reporting

The rapid review was written up into a template document developed in line with the UK NSC’s evidence summary reporting checklist [12]. The checklist specifies 16 items covering 6 key areas (title and summaries, introduction and approach, search strategy and study selection [for each question], study level reporting of results [for each question], question level synthesis, and review summary). A completed version of the reporting checklist for this rapid review can be found in the Supplementary Materials (Additional file 1). The use of a report template encourages transparency and consistency of reporting of the methods and results of each rapid review commissioned by the UK NSC on different conditions.

UK NSC evidence summaries do not extend to the conduct of a quantitative meta-analysis. Instead, for each question, relevant studies identified in the review were grouped by outcome reported and summarised narratively. Where epidemiology study results were consistent, summary ranges were presented, while the results of diagnostic test accuracy studies (in terms of sensitivity, specificity, positive and negative predictive values and accuracy) were tabulated. Reference was made to existing SLRs, and the results of a review addressing the epidemiology of VP were summarised. However, an SLR of ultrasound testing for VP was not used in this way because it did not present the results of the included studies in sufficient detail to inform an analysis of second-trimester transabdominal sonography specifically, which was required to answer the review question. Equally, an SLR relating to VCI was hand-searched but not included, as the outcomes investigated within were not searched for systematically.

The quality of the identified studies fed into a narrative analysis, which informed the conclusion of the review regarding each question. The analysis for each question was structured using themes from the Grading of Recommendations Assessment, Development and Evaluation (GRADE) framework, taking into account the volume, quality, applicability and consistency of the evidence. In some cases, appraisal of the volume of the evidence alone (in terms of the number of studies and the number of participants in each study) was sufficient to determine that a UK NSC criterion could not be met.

As well as discussing the limitations of the available evidence, e.g. in terms of evidence gaps, a key section of the report discussed the limitations of the review methodology, e.g. pertaining to eligibility criteria (such as the exclusion of congress abstracts and non-English language publications, and the use of date limits for studies on epidemiological outcomes) and the record screening approach (such as the use of a single reviewer screening records for relevance in the first instance). An assessment was made of the likely impact of any such methodological choices in terms of the likelihood of pivotal studies being missed [11]. A tabulated summary of this section has been provided in Table 3.

**Table 3:** Summary of methodological approaches taken in the rapid review and possible implications regarding the validity of the review

Methodological approach	Possible impact on validity of the review
Only including peer-reviewed journal publications, and excluding any literature that was not peer-reviewed such as congress presentations and government reports	This may have led to the exclusion of relevant evidence that has only been published in non-peer-reviewed formats. However, this is an accepted methodological adjustment for a rapid review and is unlikely to miss any pivotal studies, which would likely be published in peer-reviewed journals
Only including English language publications	Given that this review focused on evidence relevant to the UK setting, this limitation should not have led to the exclusion of any pivotal studies
Searches were run without date limits, and studies were initially considered for inclusion regardless of when they were conducted or published. However, given that a high volume of studies reporting epidemiology outcomes were identified, it was necessary to reduce the number of studies selected for extraction. Studies completed after 2000 and 2006 were ultimately included for VP and VCI respectively	Although some evidence from older studies was therefore excluded from the evidence synthesis, there is evidence that rates of VP have changed over time. The underlying risk factors for VP and VCI, particularly in vitro fertilisation(IVF), have also changed over time. The most recent estimates are therefore likely to provide the most relevant estimates of current epidemiology
Articles were reviewed by a single reviewer in the first instance. A second reviewer examined all included articles, 20% of excluded articles and any articles where there was uncertainty about inclusion	Although a systematic review would require all articles to be reviewed in duplicate to reduce the risk of bias as much as possible, this pragmatic strategy would have ensured that any articles where the eligibility was unclear were reviewed twice. Furthermore, input from clinical experts and public consultation on the results acted as a safeguard to minimise the risk of critical studies being missed
Searches for full-text articles were carried out at Cambridge University Library, but some articles were not freely available at this library and were therefore not reviewed	One article was included in the evidence synthesis on the basis of the abstract alone, but for the remainder of the articles, it was judged that the full-texts would not contain any additional pivotal data from relevant populations that would affect the conclusions of the review
Not contacting authors of publications for further information	The anticipated impact of this was expected to be minimal, especially given the small proportion of timely responses typically received when undertaking this activity. Furthermore, none of the queries would have related to a matter with the potential to change the direction of the conclusions drawn in the review
Use of published quality assessment checklists without adaptation, to minimise the time required for protocol development	This action reduced time required at the protocol development stage, but in retrospect may have increased the time taken to integrate the quality assessment results into the discussion. Nevertheless, this had no effect on the conclusions of the review

### Application of published checklists to the rapid review

To inform a discussion in this paper on different aspects of the VP evidence summary, we applied a checklist of practical items to consider when choosing a rapid review approach, as proposed by Kaltenthaler et al. [4], and the AMSTAR 2 methodological quality checklist [13]. We also applied the Preferred Reporting Items for Systematic Reviews and Meta-Analyses (PRISMA) checklist [14] to the online version of the report [11].

## Results

### Review conclusions

The evidence summary reported that little had changed since the previous review in terms of key evidence gaps relating to the epidemiology of VP, the screening test and the management pathway. As a result, the UK NSC concluded that there was insufficient evidence to support a change in its previous recommendation against screening. For VCI, there were some outcomes where the direction of the evidence could not be established due to the limitations of the evidence summary methods.

### Person-power required

The rapid review was commissioned in May 2016, protocol development commenced in late June 2016, and a first draft of the evidence summary relating to the 9 review questions was prepared in August 2016. The interval from the start of protocol development to delivery of the first draft of the evidence summary was 8 calendar weeks; over this period, approximately 30 person-days of time from the review team were required.

### Application of published checklists to the rapid review

An assessment of the review was performed against a checklist of “items to consider when determining a rapid review approach”, developed by Kaltenthaler et al. [4]. This checklist includes domains for adequate assessment of the current evidence base, presentation of the evidence, clarity of communication with policy-makers, and clarity of reporting of the rapid review methods used and the impact this may have had on the findings of the review. The current review performed well in all four domains of the checklist. Full details of the assessment are provided in the Supplementary Materials (Additional file 2).

A summary of the methodological quality assessment using the AMSTAR 2 checklist is presented in Table 4, with full details provided in the Supplementary Materials (Additional file 3). The rapid review scored well in criteria relating to the development of an a priori review protocol, the search strategy and the reporting and discussion of results. However, some of the methodological choices taken as part of the rapid approach adversely impacted on the assessment of review quality as measured by the standards of an SLR, particularly regarding the lack of duplicate performance of study selection and data extraction. Some questions additionally pertained to quantitative data synthesis, which is not performed for UK NSC evidence summaries; a decision was therefore made to consider these questions as not applicable.

**Table 4:** Summary of the AMSTAR 2 quality assessment

Question	Assessment
1. Did the research questions and inclusion criteria for the review include the components of PICO?	Yes
2. Did the report of the review contain an explicit statement that the review methods were established prior to the conduct of the review and did the report justify any significant deviations from the protocol?	Yes
3. Did the review authors explain their selection of the study designs for inclusion in the review?	Yes
4. Did the review authors use a comprehensive literature search strategy?	Yes
5. Did the review authors perform study selection in duplicate?	No
6. Did the review authors perform data extraction in duplicate?	No
7. Did the review authors provide a list of excluded studies and justify the exclusions?	Yes
8. Did the review authors describe the included studies in adequate detail?	Yes
9. Did the review authors use a satisfactory technique for assessing the risk of bias (RoB) in individual studies that were included in the review?	Yes
10. Did the review authors report on the sources of funding for the studies included in the review?	No
11. If meta-analysis was performed, did the review authors use appropriate methods for statistical combination of results?	Not applicable
12. If meta-analysis was performed, did the review authors assess the potential impact of RoB in individual studies on the results of the meta-analysis or other evidence synthesis?	Not applicable
13. Did the review authors account for RoB in individual studies when interpreting/ discussing the results of the review	Yes
14. Did the review authors provide a satisfactory explanation for, and discussion of, any heterogeneity observed in the results of the review?	Yes
15. If they performed quantitative synthesis, did the review authors carry out an adequate investigation of publication bias (small study bias) and discuss its likely impact on the results of the review?	Not applicable
16. Did the review authors report any potential sources of conflict of interest, including any funding they received for conducting the review?	Partially*

*Not explicitly reported in the online version of the report, but subsequently reported in the detailed methodology and results manuscript currently under review

Finally, detailed results of an assessment of the original review report against the PRISMA reporting checklist are provided in the Supplementary Materials (Additional file 4). As they pertained to meta-analysis, 5 of the 27 checklist items were deemed not applicable. Out of the remaining 22 checklist items, 16 were adequately met. The items that were not adequately met included the following: providing all specified details in the structured (executive) summary (as details of the review methodology were not provided, this item was deemed partially met), providing a reference to the review protocol in the report, providing details of the data extraction methodology, providing a statement of the principal summary outcome measures, providing an overall assessment of the risk of bias and reporting of the funding source for the review (as this is inferable but was not made explicit, the item was deemed only partially met).

## Discussion

The limitations of rapid review approaches in general have been discussed in detail elsewhere [3, 5, 15, 16] but in summary relate to the increased risk of bias and errors that deviations from standard SLR methodology could introduce to the review product. In certain circumstances, such as when timeframes for informed decision-making are short, these risks are deemed an acceptable trade-off in exchange for the increased efficiency of a rapid review. Nevertheless, the general limitations of rapid review methodologies, and their implications for the evidence summary under discussion here, should be borne in mind.

This paper is a case report describing the development of a single rapid review within a structured decision-making process in the UK. Assessment of a larger sample of UK NSC evidence summaries would help to position this case report in a wider context. Furthermore, the appraisals of the rapid review presented here against the Kaltenthaler et al., AMSTAR 2 and PRISMA checklists were conducted by the commissioners and reviewers responsible for the product, which introduces the potential for a conflict of interest. It should additionally be noted that the AMSTAR 2 and PRISMA checklists were not developed specifically for rapid reviews, and the checklist for a rapid review approach proposed by Kaltenthaler et al. has not been validated. As such, future appraisals with new instruments specifically tailored for rapid reviews might give different outcomes. Although these limitations of the current paper should be kept in mind, our aim was primarily to report our experience. The use of published checklists was applied to structure reflection on this experience, and similar approaches have been published previously [4].

Although not validated, Kaltenthaler et al.’s checklist of items to be considered when undertaking a rapid review [4] has been found to be a useful point of reference [17]. The UK NSC approach to the production of evidence summaries aligns closely with these recommendations. For example, with regard to understanding the existing evidence base, the current evidence summary built on the previous UK NSC reviews of screening for VP. This had several advantages, such as commissioner awareness of the evidence base and ongoing developments in the standard-setting environment. Importantly, key issues for the current review (such as the need to focus some questions on VCI) had been identified during the public consultation on the previous review. In addition, scoping searches were undertaken during development of the review brief and by the review team during preparation of the protocol. The limited volume of literature on VP and VCI enabled a straightforward search strategy and avoided the need to develop targeted approaches to each review question.

With regard to Kaltenthaler et al.’s other checklist items, the UK NSC has clearly-stated commissioning requirements for evidence summaries, a reporting checklist, and a report template requiring the methods and limitations of the evidence summary to be described. The current evidence summary was developed within a process providing opportunities for peer review, panel discussion and public comments on documents before they form the basis of a UK NSC recommendation [11]. The review team met frequently with the UK NSC commissioning team and, in turn, commissioners were in regular contact with experts in the field of obstetrics, both to ensure that no key studies had been missed from the rapid review and to solicit their input on the interpretation of evidence.

In terms of reporting, according to our assessment, the original, published write-up performed moderately against the PRISMA checklist, meeting 16 of 22 items considered relevant to a review which did not plan to undertake a meta-analysis; in Kelly et al.’s analysis of 66 rapid review products, the mean number of adequately reported PRISMA items was 14.5 for published reviews and 11.7 for unpublished reviews [5]. In terms of quality, according to our assessment, the conduct of this evidence summary evaluated moderately against the AMSTAR 2 checklist. It should be noted that the AMSTAR 2 checklist was not designed to generate an overall score [13] and questions on quantitative data synthesis were judged to be not applicable (questions 11, 12 and 15); nevertheless, the review met the majority of applicable AMSTAR 2 checklist items. This compares well with an average of 39% in Kelly et al.’s analysis [5] using the original AMSTAR checklist [18] and similarly with the quality assessment results of 3 rapid reviews reported by Kaltenthaler et al. [4], all of which adequately met 82% or more of AMSTAR checklist items. However, it should be noted that neither Kelly et al. nor Kaltenthaler et al. removed questions related to quantitative synthesis from their analysis.

The current review did not perform well in the AMSTAR 2 criteria relating to the use of duplicate study selection and data extraction, and the reporting of funding sources for included studies. These may be limitations of the evidence summary. However, use of a single reviewer screening studies for eligibility is an approach that is reported to be used in approximately half of rapid reviews [19]; when this approach is combined with verification of a subset of records by another reviewer, as performed in the current review, it has been described by the World Health Organization (WHO) as a reasonable approach to study selection in rapid reviews [19]. Similarly, the WHO identifies data extraction by a single reviewer, with or without verification, as the most common approach taken in rapid reviews, and goes on to define the use of a single reviewer extracting data with a second reviewer checking at least a 10% random sample (or, alternatively, with a focus on the quantitative results) as a reasonable approach [19]. As previously described, the current review used a single reviewer for data extraction, with a second reviewer verifying all extracted data. Furthermore, the UK NSC uses several additional safeguards to minimise the risks of missing critical studies, including input from clinical experts and public consultation on the review.

In keeping with UK NSC requirements for evidence summaries, a meta-analysis was not performed in the current review, and this approach to data synthesis was specified a priori. This prevented an estimate being made of the direction of the evidence for some outcomes relating to VCI. Despite this, and despite other shortcomings of the review as identified through application of the AMSTAR 2 checklist, the evidence summary approach identified significant concerns about the volume and quality of evidence relating to key questions on the UK epidemiology of VP, the test and the management of the condition.

The use of narrative synthesis in the current review is in keeping with the results of Kelly et al.’s analysis, which reported that 62/66 rapid reviews (94%) did not attempt meta-analysis. In the current review, narrative synthesis enabled the identification of a subset of outcomes for which focused meta-analyses may help to address conflicting reports of associations between VCI and fetal/neonatal mortality, pre-eclampsia and low Apgar score. The narrative synthesis also highlighted the paucity of prospective research into the benefits and harms of defined screening, management and intervention pathways. As a result of this, two related projects have been commissioned. These are a meta-analysis to explore the subset of issues relating to VCI and a modelling exercise to estimate the potential impact of screening and help gauge the viability of primary research. In this sense, the review met the commissioning requirement to highlight further work that could be done before the next scheduled review of this topic.

As the current rapid review was considered to be an adequate tool for the UK NSC decision-making mechanism but did not meet all relevant AMSTAR 2 checklist items, this poses the question of how to measure the quality of rapid review products. SLR methods provide a valuable reference point for the conduct and reporting of rapid reviews and serve to identify factors potentially increasing the risk of bias in rapid reviews as compared with SLRs. However, SLR quality appraisal tools may undervalue rapid reviews if the purpose and context of these products are not factored in to the assessment of quality. None of the checklists applied in the current case report considered these elements, which may be beneficial in any future quality assessment and reporting checklists being developed and validated for use specifically on rapid reviews.

## Conclusions

In this case report, the rapid review results were available within the required timelines and enabled the UK NSC to make an informed decision about whether there was sufficient evidence to reconsider the established recommendation regarding population screening for VP. On this occasion, use of a rapid review as part of the UK NSC’s process enabled a pragmatic approach to assessing the overall volume, quality and direction of literature on key questions relating to the viability of a population screening programme for VP. Confirmation of the validity of the methods used in this case report, as well as the wider generalisability of our insights relating to rapid review practice, reporting and quality assessment, would require analysis of a larger sample of UK NSC reviews.

## Supplementary information

**Additional file 1: Table S1.** UK NSC reporting checklist for evidence summaries, completed for the VP review. This table contains a completed version of the reporting checklist for the rapid review.

**Additional file 2: Table S2.** Quality assessment against checklist adapted from Kaltenthaler et al. This table contains full details of the results of the quality assessment of the rapid review using the checklist developed by Kaltenthaler et al.

**Additional file 3: Table S3.** AMSTAR 2 quality assessment of the evidence summary. This table contains full details of the results of the quality assessment of the rapid review using the AMSTAR 2 checklist.

**Additional file 4: Table S4.** Completed PRISMA checklist for the rapid review.

## Data Availability

The rapid review on screening for vasa praevia conducted for the UK NSC is available on the Legacy Screening website (https://legacyscreening.phe.org.uk/vasapraevia). A manuscript reporting results of the rapid review is under review.

## Abbreviations

CS: Caesarean section
GRADE: Grading of Recommendations Assessment, Development and Evaluation
IVF: In vitro fertilisation
NHS: National Health Service
NICE: National Institute for Health and Care Excellence
NSC: National Screening Committee
PICO: Population, Intervention, Comparator and Outcomes
PRISMA: Preferred Reporting Items for Systematic Reviews and Meta-Analyses
RoB: Risk of bias
SLR: Systematic literature review
VCI: Velamentous cord insertion
VP: Vasa praevia
WHO: World Health Organization

## Footnotes

1 National Institute for Health and Care Excellence (NICE) definition of “evidence summary”: “Produced following a clear and transparent process in order to summarise the best available evidence on a topic. Does not meet the full requirements for a systematic review in that the inclusion criteria may be less comprehensive (for example by time period, type of evidence or exhaustivity of search/selection). It may or may not include an evidence synthesis”.
