# Supplementary material for: Case report: a rapid review approach used by the UK National Screening Committee to inform recommendations on general population screening for vasa praevia
Source: Syst Rev. 2019 Dec 29;8:340. doi: 10.1186/s13643-019-1244-9 (PMC6935491; doi:10.1186/s13643-019-1244-9)
Supplement: Supplementary file 1 — Additional file 1: Table S1. UK NSC reporting checklist for evidence summaries, completed for the VP review. This table contains a completed version of the reporting checklist for the rapid review. [file 13643_2019_1244_MOESM1_ESM.docx]

**Additional file 1: Table S1. UK NSC reporting checklist for evidence summaries,* completed for the online version of the VP rapid review report****

|  | Section | Item | Page no. |
| --- | --- | --- | --- |
| 1. | TITLE AND SUMMARIES | | |
| 1.1 | Title sheet | Identify the review as a UK NSC evidence summary. | Title page |
| 1.2 | Plain English summary | Plain English description of the executive summary. | 1 |
| 1.3 | Executive summary | Structured overview of the whole report. To include: the purpose/aim of the review; background; previous recommendations; findings and gaps in the evidence; recommendations on the screening that can or cannot be made on the basis of the review. | 1 |
| 2. | INTRODUCTION AND APPROACH | | |
| 2.1 | Background and objectives | Background – Current policy context and rationale for the current review – for example, reference to details of previous reviews, basis for current recommendation, recommendations made, gaps identified, drivers for new reviews  Objectives – What are the questions the current evidence summary intends to answer? – statement of the key questions for the current evidence summary, criteria they address, and number of studies included per question, description of the overall results of the literature search.  Method – briefly outline the rapid review methods used. | 8 |
| 2.2 | Eligibility for inclusion in the review | State all criteria for inclusion and exclusion of studies to the review clearly (PICO, dates, language, study type, publication type, publication status etc.) To be decided *a priori*. | 47 |
| 2.3 | Appraisal for quality/risk of bias tool | Details of tool/checklist used to assess quality, for example QUADAS 2, CASP, SIGN, AMSTAR. | 133 |
| 3. | SEARCH STRATEGY AND STUDY SELECTION (FOR EACH KEY QUESTION) | | |
| 3.1 | Databases/ sources searched | Give details of all databases searched (including platform/interface and coverage dates) and date of final search. | 46 |
| 3.2 | Search strategy and results | Present the full search strategy for at least one database (usually a version of Medline), including limits and search filters if used.  Provide details of the total number of (results from each database searched), number of duplicates removed, and the final number of unique records to consider for inclusion. | 46 |
| 3.3 | Study selection | State the process for selecting studies – inclusion and exclusion criteria, number of studies screened by title/abstract and full text, number of reviewers, any cross checking carried out. | 47 |
| 4. | STUDY LEVEL REPORTING OF RESULTS (FOR EACH KEY QUESTION) | | |
| 4.1 | Study level reporting, results and risk of bias assessment | For each study, produce a table that includes the full citation and a summary of the data relevant to the question (for example, study size, PICO, follow-up period, outcomes reported, statistical analyses etc.).  Provide a simple summary of key measures, effect estimates and confidence intervals for each study where available.  For each study, present the results of any assessment of quality/risk of bias. | Study level reporting: 59  Quality assessment: 134 |
| 4.2 | Additional analyses | Describe additional analyses (for example, sensitivity, specificity, PPV, etc.) carried out by the reviewer. | Study level analyses within data extraction tables: 59 |
| 5. | QUESTION LEVEL SYNTHESIS | | |
| 5.1 | Description of the evidence | For each question, give numbers of studies screened, assessed for eligibility, and included in the review, with summary reasons for exclusion. | 12 |
| 5.2 | Combining and presenting the findings | Provide a balanced discussion of the body of evidence which avoids over reliance on one study or set of studies. Consideration of four components should inform the reviewer’s judgement on whether the criterion is ‘met’, ‘not met’ or ‘uncertain’: quantity; quality; applicability and consistency. | 12 |
| 5.3 | Summary of findings | Provide a description of the evidence reviewed and included for each question, with reference to their eligibility for inclusion.  Summarise the main findings including the quality/risk of bias issues for each question.  Have the criteria addressed been ‘met’, ‘not met’ or ‘uncertain’? | 12 |
| 6. | REVIEW SUMMARY | | |
| 6.1 | Conclusions and implications for policy | Do findings indicate whether screening should be recommended?  Is further work warranted?  Are there gaps in the evidence highlighted by the review? | 42 |
| 6.2 | Limitations | Discuss limitations of the available evidence and of the review methodology if relevant. | 44 |
| * Public Health England. Appendix H: Reporting checklist for UK NSC evidence summaries. 2015. [Accessed: 25th March 2019] [Available from: https://www.gov.uk/government/publications/uk-nsc-evidence-review-process].  **UK National Screening Committee. 2017. Screening for Vasa Praevia in the Second Trimester of Pregnancy [Accessed: 29th July 2019] [Available from: <https://legacyscreening.phe.org.uk/vasapraevia>]. | | | |
