# Supplementary material for: Case report: a rapid review approach used by the UK National Screening Committee to inform recommendations on general population screening for vasa praevia
Source: Syst Rev. 2019 Dec 29;8:340. doi: 10.1186/s13643-019-1244-9 (PMC6935491; doi:10.1186/s13643-019-1244-9)
Supplement: Supplementary file 2 — Additional file 2: Table S2. Quality assessment against checklist adapted from Kaltenthaler et al. This table contains full details of the results of the quality assessment of the rapid review using the checklist developed by Kaltenthaler et al. [file 13643_2019_1244_MOESM2_ESM.docx]

**Additional file 2: Table S2. Assessment against checklist developed by Kaltenthaler et al.* when determining a rapid review approach**

| **Checklist item** | **Assessment** |
| --- | --- |
| 1. Assess the current evidence base. It is important to have an understanding of the evidence available before deciding which rapid review methods are most appropriate. Consider:   - Scoping searches – these are useful to estimate an approximate number of anticipated relevant studies. - Existing systematic reviews – what are the search dates for the review(s) and the question answered by the review(s)? What is the methodological quality of the review(s)? This can be assessed using appropriate checklists. Did the review report a quality assessment of included studies? Consider using reported data to incorporate in a meta-analysis with newly identified studies. - Summary of existing reviews – the findings of identified reviews could be presented plus a summary of any new studies using narrative synthesis. | Yes. Development of the review questions was informed by previous UK NSC reviews of screening for VP, while scoping searches informed the search strategy and identified published, potentially relevant SLRs that could be utilised in the current review |
| 2. Consider presentation of the evidence. The complexity of the evidence base should be taken into account and an assessment made as to how much data should be presented and in what format. Consider:   - Meta-analysis – does the data support the use of meta-analysis? - Outcome data – can limited data on outcomes be reported? - Grouping of outcomes – can relevant outcomes be grouped to assist the reader in understanding the evidence base? | Yes. Meta-analysis is not attempted in UK NSC evidence summaries, but outcomes of interest were specified *a priori* and a narrative synthesis approach was planned at the protocol development stage |
| 3. Ensure clear communication with policy makers. It is important that there is a common understanding between reviewers and policy makers as to the purpose of the review and the questions to be answered. Consider:   - In depth analysis – is it preferable to the policy maker to present an in depth analysis of a smaller selection of studies? - Brief overview – is it preferable to the policy maker to present less information from a wider range of studies? - Highlight gaps in the evidence – will it be helpful to the policy maker to highlight gaps in the evidence to inform future research? | Yes. Regular meetings between the review team and the commissioning team enabled the commissioning team to contribute to the review protocol, study selection and drafts of the report. Topic area expertise was available as required. A subsection of the “review summary” section of the report covered the identification of any relevant evidence gaps |
| 4. Clearly report rapid review methods used. It is crucial that the reader understands what rapid review methods have been used and the impact this may have on the findings of the review. Consider:   - Description of methods – have the rapid review methods been transparently reported highlighting differences from standard systematic review methods? - Discussion of limitations – have the potential limitations and biases of chosen methods been described. | Yes. Review methods, including search terms, were reported reproducibly and a subsection of the “review summary” section of the report covered limitations of the review methods and the likely impact of these limitations on the ability of the review to identify key studies |

* Kaltenthaler E, Cooper K, Pandor A, Martyn-St James M, Chatters R, Wong R. The use of rapid review methods in health technology assessments: 3 case studies. BMC Med Res Methodol. 2016;16(1):108.
