# Supplementary material for: Case report: a rapid review approach used by the UK National Screening Committee to inform recommendations on general population screening for vasa praevia
Source: Syst Rev. 2019 Dec 29;8:340. doi: 10.1186/s13643-019-1244-9 (PMC6935491; doi:10.1186/s13643-019-1244-9)
Supplement: Supplementary file 3 — Additional file 3: Table S3. AMSTAR 2 quality assessment of the evidence summary. This table contains full details of the results of the quality assessment of the rapid review using the AMSTAR 2 checklist. [file 13643_2019_1244_MOESM3_ESM.docx]

**Additional file 3: Table S3. AMSTAR 2 quality assessment of the evidence summary**

| **Question** | **Assessment** | **Justification** |
| --- | --- | --- |
| 1. Did the research questions and inclusion criteria for the review include the components of PICO? | Yes | A detailed review protocol was developed a priori, specifying the search strategy, eligibility criteria (in PICO format), record review process, and the approach to data extraction and quality assessment |
| 2. Did the report of the review contain an explicit statement that the review methods were established prior to the conduct of the review and did the report justify any significant deviations from the protocol? | Yes |  |
| 3. Did the review authors explain their selection of the study designs for inclusion in the review? | Yes | The hierarchical approach planned with regard to the inclusion of different study design types was clearly explained in the eligibility criteria, as was the fact that only published literature was eligible for inclusion (non-peer-reviewed publication types, such as congress abstracts, were excluded) |
| 4. Did the review authors use a comprehensive literature search strategy? | Yes | A sensitive search of published literature was conducted in the major electronic databases (MEDLINE, Embase and the Cochrane Library) and clearly reported. Grey literature was not searched |
| 5. Did the review authors perform study selection in duplicate? | No | Each record was reviewed against the eligibility criteria by one reviewer, with a second reviewer providing input in cases of uncertainty and validating 20% of the first reviewer’s screening decisions |
| 6. Did the review authors perform data extraction in duplicate? | No | The extraction of data from included studies was performed by a single individual for each included study. When the initial extraction was complete, a second individual independently verified the extracted information and checked that no relevant information had been missed |
| 7. Did the review authors provide a list of excluded studies and justify the exclusions? | Yes | Lists of studies included and excluded during the full-text review stage were provided, along with the rationales for exclusion |
| 8. Did the review authors describe the included studies in adequate detail? | Yes | Data extraction tables summarising characteristics of the included studies were provided |
| 9. Did the review authors use a satisfactory technique for assessing the risk of bias (RoB) in individual studies that were included in the review? | Yes | Quality assessments of the included studies were undertaken and reported |
| 10. Did the review authors report on the sources of funding for the studies included in the review? | No | Funding sources for included studies were not reported |
| 11. If meta-analysis was performed did the review authors use appropriate methods for statistical combination of results? | No/not applicable | UK NSC evidence summaries do not attempt meta-analysis |
| 12. If meta-analysis was performed, did the review authors assess the potential impact of RoB in individual studies on the results of the meta-analysis or other evidence synthesis? | No/not applicable | UK NSC evidence summaries do not attempt meta-analysis |
| 13. Did the review authors account for RoB in individual studies when interpreting/ discussing the results of the review | Yes | Study quality was considered as part of the framework for narrative analysis of the review results for each UK NSC criterion |
| 14. Did the review authors provide a satisfactory explanation for, and discussion of, any heterogeneity observed in the results of the review? | Yes | Consistency across the evidence base was one of the domains considered in the evidence summary |
| 15. If they performed quantitative synthesis did the review authors carry out an adequate investigation of publication bias (small study bias) and discuss its likely impact on the results of the review? | No/not applicable | UK NSC evidence summaries do not attempt meta-analysis |
| 16. Did the review authors report any potential sources of conflict of interest, including any funding they received for conducting the review? | Partly | Not reported in the online version of the report, but subsequently reported in the detailed methodology and results manuscript currently under review |
