# Supplementary material for: Case report: a rapid review approach used by the UK National Screening Committee to inform recommendations on general population screening for vasa praevia
Source: Syst Rev. 2019 Dec 29;8:340. doi: 10.1186/s13643-019-1244-9 (PMC6935491; doi:10.1186/s13643-019-1244-9)
Supplement: Supplementary file 4 — Additional file 4: Table S4. Completed PRISMA checklist for the rapid review. [file 13643_2019_1244_MOESM4_ESM.docx]

**Additional file 4: Table S4. Completed PRISMA checklist* for the online version of the VP rapid review report****

| **Section/topic** | **#** | **Checklist item** | **Reported on page #** |
| --- | --- | --- | --- |
| **TITLE** | | |  |
| Title | 1 | Identify the report as a systematic review, meta-analysis, or both. | Page 2: Identified as a rapid review in first sentence of "Executive Summary" |
| **ABSTRACT** | | |  |
| Structured summary | 2 | Provide a structured summary including, as applicable: background; objectives; data sources; study eligibility criteria, participants, and interventions; study appraisal and synthesis methods; results; limitations; conclusions and implications of key findings; systematic review registration number. | Pages 2–4: There is no requirement for an abstract in the UK NSC evidence summary template or the UK NSC evidence summary reporting checklist, but the following structure was used for the Executive Summary: Purpose of the Review; Background; Previous Recommendation; Findings and Gaps in the Evidence; Recommendations on Screening; Limitations. Details of Methods were not provided |
| **INTRODUCTION** | | |  |
| Rationale | 3 | Describe the rationale for the review in the context of what is already known. | Pages 8–10 |
| Objectives | 4 | Provide an explicit statement of questions being addressed with reference to participants, interventions, comparisons, outcomes, and study design (PICOS). | Pages 10–11 |
| **METHODS** | | |  |
| Protocol and registration | 5 | Indicate if a review protocol exists, if and where it can be accessed (e.g., Web address), and, if available, provide registration information including registration number. | Although a review protocol was developed, this was not indicated in the report |
| Eligibility criteria | 6 | Specify study characteristics (e.g., PICOS, length of follow-up) and report characteristics (e.g., years considered, language, publication status) used as criteria for eligibility, giving rationale. | Pages 47–50 |
| Information sources | 7 | Describe all information sources (e.g., databases with dates of coverage, contact with study authors to identify additional studies) in the search and date last searched. | Page 46 |
| Search | 8 | Present full electronic search strategy for at least one database, including any limits used, such that it could be repeated. | Page 46 |
| Study selection | 9 | State the process for selecting studies (i.e., screening, eligibility, included in systematic review, and, if applicable, included in the meta-analysis). | Page 47 |
| Data collection process | 10 | Describe method of data extraction from reports (e.g., piloted forms, independently, in duplicate) and any processes for obtaining and confirming data from investigators. | Not reported [though extraction methodology was pre-specified in the protocol as double reviewer] |
| Data items | 11 | List and define all variables for which data were sought (e.g., PICOS, funding sources) and any assumptions and simplifications made. | Pages 10–11 (the key questions in Table 1 are the variables for which data were sought) |
| Risk of bias in individual studies | 12 | Describe methods used for assessing risk of bias of individual studies (including specification of whether this was done at the study or outcome level), and how this information is to be used in any data synthesis. | Page 50 |
| Summary measures | 13 | State the principal summary measures (e.g., risk ratio, difference in means). | Outcomes were defined in PICOS tables (pages 47–50), although no measures were specifically reported as principal |
| Synthesis of results | 14 | Describe the methods of handling data and combining results of studies, if done, including measures of consistency (e.g., I^2^) for each meta-analysis. | Narrative synthesis was planned and conducted; meta-analysis was therefore N/A and as such was not described |
| Risk of bias across studies | 15 | Specify any assessment of risk of bias that may affect the cumulative evidence (e.g., publication bias, selective reporting within studies). | No overall assessment of the risk of bias was reported |
| Additional analyses | 16 | Describe methods of additional analyses (e.g., sensitivity or subgroup analyses, meta-regression), if done, indicating which were pre-specified. | Narrative synthesis was planned and conducted; meta-analysis was therefore N/A and as such was not described |
| **RESULTS** | | | |
| Study selection | 17 | Give numbers of studies screened, assessed for eligibility, and included in the review, with reasons for exclusions at each stage, ideally with a flow diagram. | Page 12; page 51 |
| Study characteristics | 18 | For each study, present characteristics for which data were extracted (e.g., study size, PICOS, follow-up period) and provide the citations. | Pages 59–133 |
| Risk of bias within studies | 19 | Present data on risk of bias of each study and, if available, any outcome level assessment (see item 12). | Pages 134–139 |
| Results of individual studies | 20 | For all outcomes considered (benefits or harms), present, for each study: (a) simple summary data for each intervention group (b) effect estimates and confidence intervals, ideally with a forest plot. | Pages 59–133 |
| Synthesis of results | 21 | Present results of each meta-analysis done, including confidence intervals and measures of consistency. | Narrative synthesis was planned and conducted; meta-analysis was therefore N/A |
| Risk of bias across studies | 22 | Present results of any assessment of risk of bias across studies (see Item 15). | N/A |
| Additional analysis | 23 | Give results of additional analyses, if done (e.g., sensitivity or subgroup analyses, meta-regression [see Item 16]). | Narrative synthesis was planned and conducted; meta-analysis was therefore N/A |
| **DISCUSSION** | | | |
| Summary of evidence | 24 | Summarize the main findings including the strength of evidence for each main outcome; consider their relevance to key groups (e.g., healthcare providers, users, and policy makers). | Pages 12–41 |
| Limitations | 25 | Discuss limitations at study and outcome level (e.g., risk of bias), and at review-level (e.g., incomplete retrieval of identified research, reporting bias). | Pages 43–45 |
| Conclusions | 26 | Provide a general interpretation of the results in the context of other evidence, and implications for future research. | Pages 42–44 |
| **FUNDING** | | | |
| Funding | 27 | Describe sources of funding for the systematic review and other support (e.g., supply of data); role of funders for the systematic review. | Title subheading on the cover page states the external review was conducted for the UK NSC, but the funding source was not explicitly stated |
| * Moher D, Liberati A, Tetzlaff J, Altman DG, The PRISMA Group (2009). Preferred Reporting Items for Systematic Reviews and Meta-Analyses: The PRISMA Statement. PLoS Med 6(7): e1000097. doi:10.1371/journal.pmed1000097.  **UK National Screening Committee. 2017. Screening for Vasa Praevia in the Second Trimester of Pregnancy [Accessed: 29th July 2019] [Available from: <https://legacyscreening.phe.org.uk/vasapraevia>]. | | | |
